# Novel TiO_2_/GO/M-MMT nano-heterostructured composites exhibiting high photocatalytic activity

**DOI:** 10.3389/fchem.2023.1113186

**Published:** 2023-03-09

**Authors:** W. Li, Y. He, W. B. Bao, H. L. Bao, D. Y. Li, C. L. Zhang, M. Wang

**Affiliations:** ^1^ School of Architecture and Civil Engineering, Shenyang University of Technology, Shenyang, Liaoning, China; ^2^ School of Materials Science and Engineering, Liaoning Technical University, Fuxin, Liaoning, China

**Keywords:** TiO_2_/GO/M-MMT composite, methyl orange, photocatalytic performance, electron transfer ability, nanoheterostructures

## Abstract

This study proposed a technique to enhance the photocatalytic properties of TiO_2_ using graphene oxide (GO) and modified Montmorillonite (M-MMT). TiO_2_/GO/M-MMT nano-heterostructured composites were prepared *via* hydrothermal and co-precipitation. The photocatalytic performance was evaluated by investigating the photodegradation rate and absorption behavior of methyl orange (MO) under visible light irradiation. The results showed that TiO_2_/GO/M-MMT heterojunction exhibited excellent photocatalytic degradation performance, as the degradation rate of MO was observed to be 99.3% within 150 min. The density of adsorbed MO decreased by 62.1% after 210 min of dark adsorption using the TiO_2_/GO/M-MMT composite, which was significantly higher than that achieved using M-MMT, GO/M-MMT, and TiO_2_/M-MMT. The nano-heterostructure increased the effective interface between TiO_2_, GO, and MMT, which increased the charge transfer ability and prolonged the electron-hole separation time. Therefore, the results of this study can be used to design novel photocatalysts to eradicate environmental pollutants.

## 1 Introduction

Environmental pollution engendered from rapid advancements in the modern chemical industry has attracted the attention of researchers worldwide. Secondary chemical contamination can be caused during the degradation of pollutants using chemical methods (Laysandra et al., 2017). Photocatalytic degradation has been used to eradicate organic pollutants from wastewater owing to its several advantages such as operation simplicity, low cost and pollution-free nature (Lee et al., 2018; El-Kousy et al., 2020; Dao et al., 2021; Liu et al., 2022).

Titanium dioxide (TiO_2_) is one of the most efficient photocatalysts used to produce hydrogen owing to its low cost, high stability, corrosion resistance, and environmental friendliness (Tao et al., 2020; Wang et al., 2021). TiO_2_ photocatalysts have been used for industrial wastewater treatment using solar energy by converting the wastewater into non-toxic chemical products without producing any other pollutants. However, because of the wide band gap, TiO_2_ must be irradiated with ultraviolet light, which constitutes approximately only 4% of visible light and provides low quantum yield (Wang et al., 2019). TiO_2_ activity can be enhanced by coupling it with other semiconductors or doping with higher work-function metals (Yadav and Ahmad, 2015; Umer et al., 2019; Yang et al., 2020; Xiang et al., 2021; Li et al., 2022a; Dong et al., 2022; Jing et al., 2022; Xu and Li, 2022). Montmorillonite (MMT) is the most widely used material in clay semiconductor nanocomposites owing to its layered structure, high cation exchange capacity, excellent charge trapping ability, and considerable adsorption potential for semiconductor particles (Wang et al., 2011; Paiva et al., 2014; Sharma et al., 2018). Clay-TiO_2_/MMT heterostructured composites can provide additional number of sites for trapping photo-generated electrons, which ultimately enhances the photocatalytic activity (Liao et al., 2022; Wang et al., 2022). Additionally, graphene oxide (GO) can enhance the catalytic effect of semiconductors (Joshi et al., 2020) and can be used to provide electric carriers with more active attachment-sites for photocatalysis. This enables a faster transfer of photoelectrons, avoids accumulation because of its high electrical conductivity and large specific surface area, thereby reducing the possibility of electron-hole recombination. Liu et al. (2021) prepared N-TiO_2_/GO photocatalyst *via* sol-gel and hydrothermal methods and analyzed its adsorption performance for RhB.

Photocatalysis is a new, efficient and potential discovery. It uses renewable energy to decompose organic pollutants by sunlight. At present, the known photocatalytic materials are semiconductor materials and polymer materials. In the past decade, polymer photocatalysts have been developed rapidly, and many polymer photocatalysts with catalytic activity have been found (Kumar et al., 2021; Nadali et al., 2021; Yin et al., 2022). However, polymer photocatalysts have limited photochemical stability, lack of understanding of the reaction mechanism, balance between charge carrier lifetime and catalytic time, and the use of unsustainable sacrificial reagents (Tahir, 2017; Dao et al., 2021). However, the photocatalytic performance of TiO_2_/GO/MMT nanoheterostructured composite has not been reported thus far. In this study, a TiO_2_/GO/MMT nanocomposite was synthesized *via* hydrothermal and co-precipitation methods and the photodegradation of methyl orange (MO) using the nanocomposite was studied.

## 2 Experimental process

Modified MMT (M-MMT; 1 g) and cetyltrimethylammonium bromide (CTAB; 1 g) (∼1% of MMT mass) were added to distilled water (200 mL) under ultrasonic conditions for 60 min. A certain amount of GO [prepared *via* the modified Hummer method (Pu et al., 2019)] was added to the M-MMT solution and stirred for 12 h using a magnetic stirrer. The solution was kept idle at room temperature for 24 h. Subsequently, the precipitates were washed, dried, and grinded to obtain the GO/M-MMT composite.

CTAB and butyl titanate were weighed at a ratio of 1:2 (n_(CTAB)_/n_(Ti)_ = 0.5). The CTAB was then dispersed in 30 mL of distilled water and butyl titanate was placed in a beaker. A certain amount of the GO/M-MMT composite was added to the CTAB solution. After stirring for 30 min, butyl titanate was dropped into the solution at a rate of 0.5 drop/s using a dropper. After titration, the as-prepared solution was poured into a reactor and hydrothermally treated at 180°C for 10 h. A white powder was obtained after filtration and drying. The composite, TiO_2_/GO/M-MMT, was obtained by calcining the powder at 300°C for 50 min in a muffle furnace.

The microstructure of the TiO_2_/GO/M-MMT composite was analyzed *via* scanning electron microscopy (SEM) (JSM-7500F) and X-ray diffraction (XRD) (Shimadzu 6100). The atom bonding situation, specific surface area, and pore distribution of the TiO_2_/M-MMT, TiO_2_/GO, and TiO_2_/GO/M-MMT composites were measured using X-ray photoelectron spectroscopy (XPS) (Shimadzu/Kratos Axis Ultra DLD) and the BET (ASAP 2460) method.

The irradiation light power is 1380 W/m^2^. The wavelength range of visible light is 400–760 nm. Visible light is used to study the photocatalytic performance in the experiment. The rate of photodegradation was tested using a 722S visible spectrophotometer. The photocatalytic performance of the composite was evaluated directly by measuring the change in the rate of degradation MO. The initial absorbance of MO (denoted by A_0_) without the catalyst before illumination was determined by adjusting the wavelength. First, the suspension was stirred in dark for 60 min to achieve an adsorption–desorption equilibrium, and the solution was sampled every 20 min during the experiment. Next, the samples were centrifuged for 5 min at a speed of 4000 rpm using a high-speed centrifuge, following which the supernatant was collected to measure the absorbance (denoted by A). The following equation (Eq. 1) was used to calculate the degradation rate (*η*).

$$\eta =\frac{A_{0}-A}{A_{0}}\times 100\%$$ (1)

The composite (0.05 g) was placed in a MO solution (100 mL, 10 mg/L). The solution was irradiated with ultraviolet light and stirred in a dark box for 40 min. After centrifugation, the absorbance of the MO solution was measured at a wavelength of 460 nm. The amount of MO adsorbed by the composite was calculated using the following equation (Eq. 2).

$$q_{t}=\frac{\left(C_{0}-C_{t}\right)V}{W}$$ (2)

where $q_{t},V,and\;W$ represent the amount of MO adsorbed (mg/g), initial volume of the MO solution (L), and the quantity of the composite (g), respectively. C_0_ and C_t_ denote the initial density (mg/L^3^) and the concentration of the MO solution (mg/L) at time t, respectively.

The activity of TiO2/GO/M-MMT photocatalyst will be evaluated by the change of methyl orange concentration and its adsorption capacity. At the same time, the pseudo-first-order kinetic equation will be given to further study the kinetics of photocatalytic degradation, and the mechanism of photocatalytic degradation will be discussed.

## 3 Results and discussion

**FIGURE 1:**
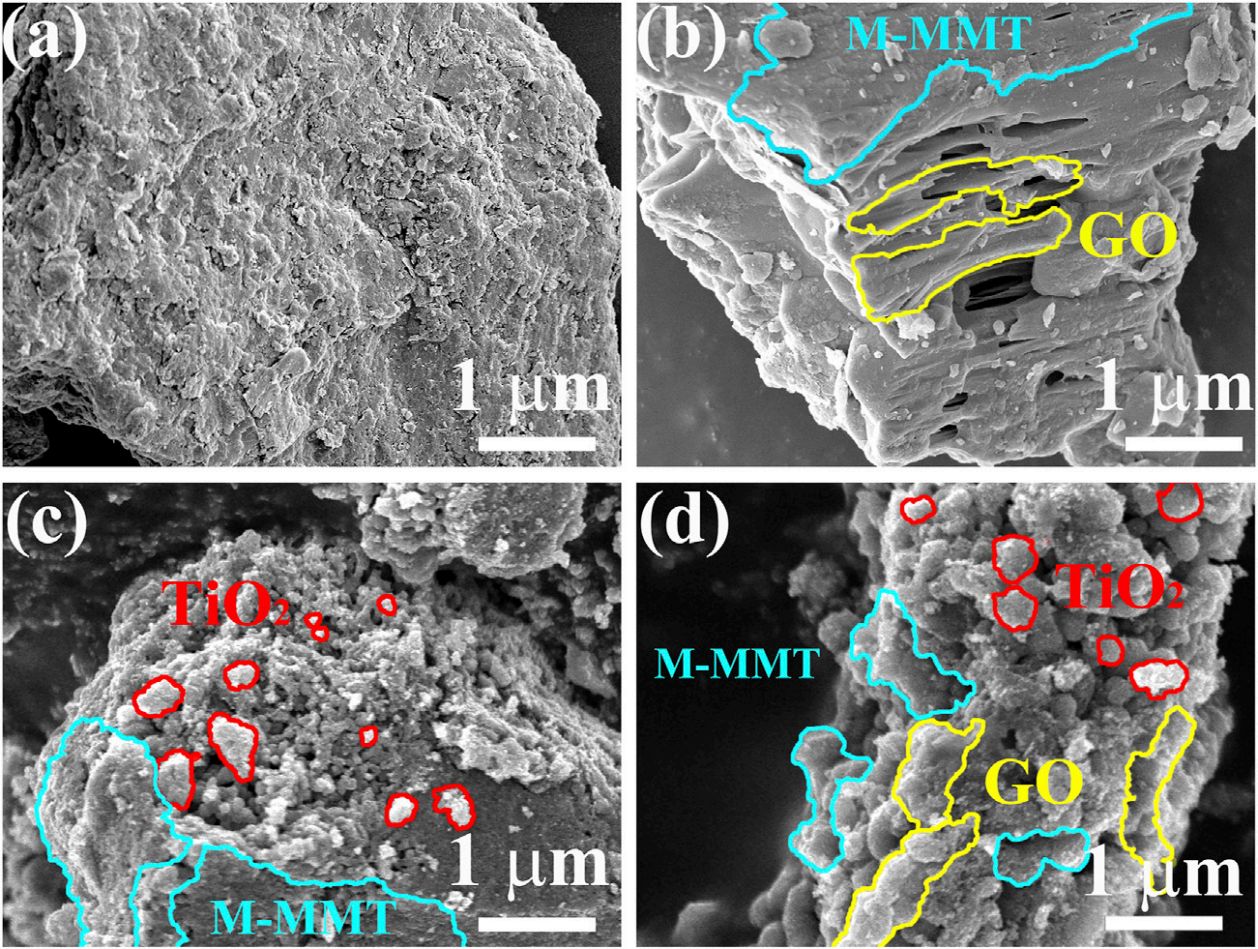
Scanning electron microscope images of different specimen. **(A)** M-MMT; **(B)** GO/M-MMT; **(C)** TiO_2_/M-MMT; **(D)** TiO_2_/GO/M-MMT.

Figure 1A shows the image of M-MMT, which displays a layered structure. Figure 1B shows the morphology of the GO/M-MMT composite. Several layers of the GO sheets are rough and wrinkled, which might provide more active sites for other components (TiO_2_). As can be observed from Figure 1C, several small and uniform TiO_2_ particles are formed on the surface of M-MMT, which is expected to improve the catalytic performance of the composite. The SEM images of the TiO_2_/GO/M-MMT nanocomposite (Figure 1D) reveals that several uniform-sized TiO_2_ nano-particles and layered GO sheets are formed on the M-MMT surface.

The energy dispersive X-ray spectroscopy results for the TiO_2_/GO/M-MMT nanocomposite (Supplementary Figure S1) reveal that Al, Si, C, O, and Ti are distributed on the TiO_2_/GO/M-MMT nanocomposite, indicating that TiO_2_, GO, and M-MMT form a uniform compound structure. The composition of the composite is illustrated further in the inset of Supplementary Figure S1B. The amounts of O, C, Si, Al, and Ti are reported as 21.51%, 73.00%, 0.06%, 0.05%, and 5.38%, respectively.

**FIGURE 2:**
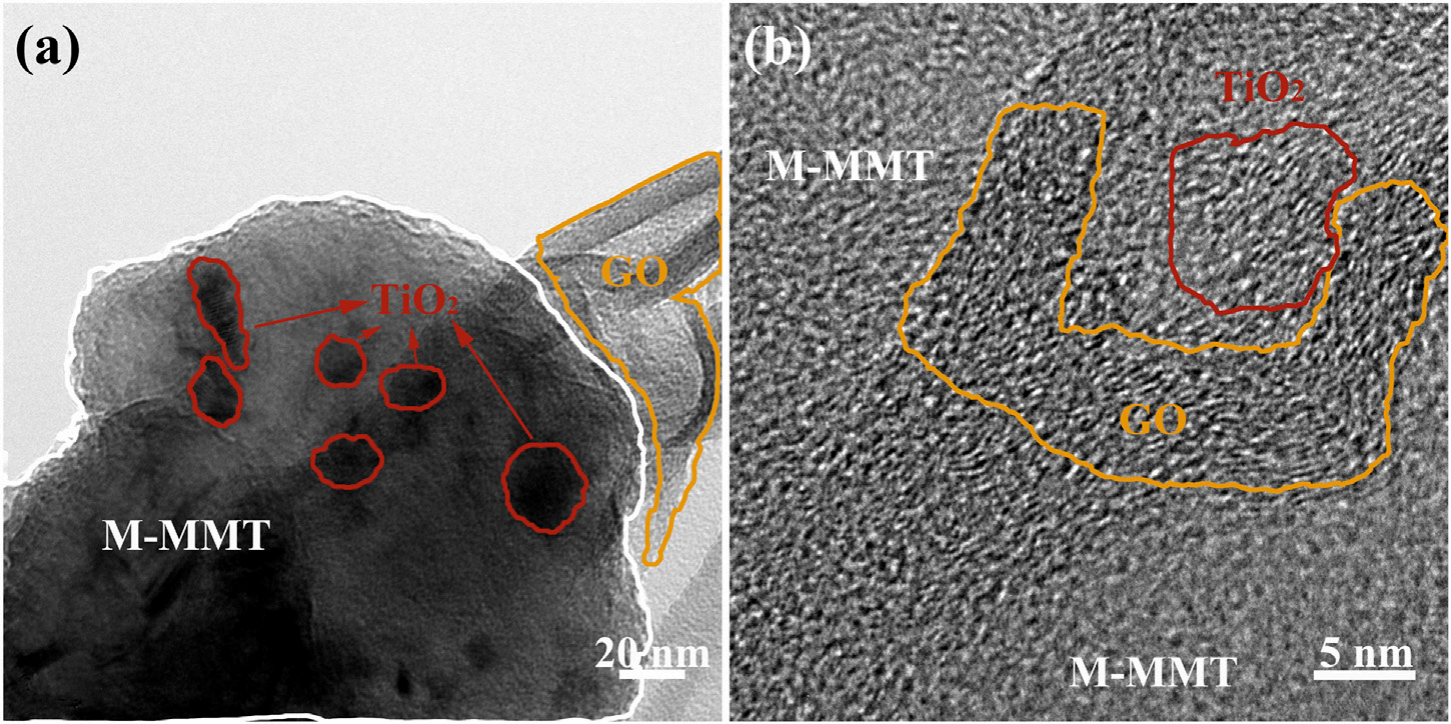
**(A)** The TEM images of TiO_2_/GO/M-MMT nanocomposites; **(B)** The HRTEM image of TiO_2_/GO/M-MMT composites.

The TEM images of TiO_2_/GO/M-MMT nanocomposites in Figure 2A, GO is circled by the orange ring, M-MMT is circled by the white line. The growth on M-MMT is TiO_2_, Which marked with a red circle. In the figure, it can be seen that the large layer M-MMT and TiO_2_ particles was tightly wraped with the film GO, and the TiO_2_ particles with a diameter of 15–20 nm grow uniformly on the M-MMT, which is consistent with the SEM in Figure 1D. The HRTEM image of TiO_2_/GO/M-MMT composites is shown in Figure 2B, in which the lattice fringes of TiO_2_ and GO can be clearly detected. The d spacing of GO is 0.38 nm, which is wider than that of Graphene. The reason for this phenomenon is that oxygen enters the interlayer of graphene, increasing the distance between the surfaces of graphene. The d spacing of TiO_2_ is 0.35 nm, corresponding to the (101).

**FIGURE 3:**
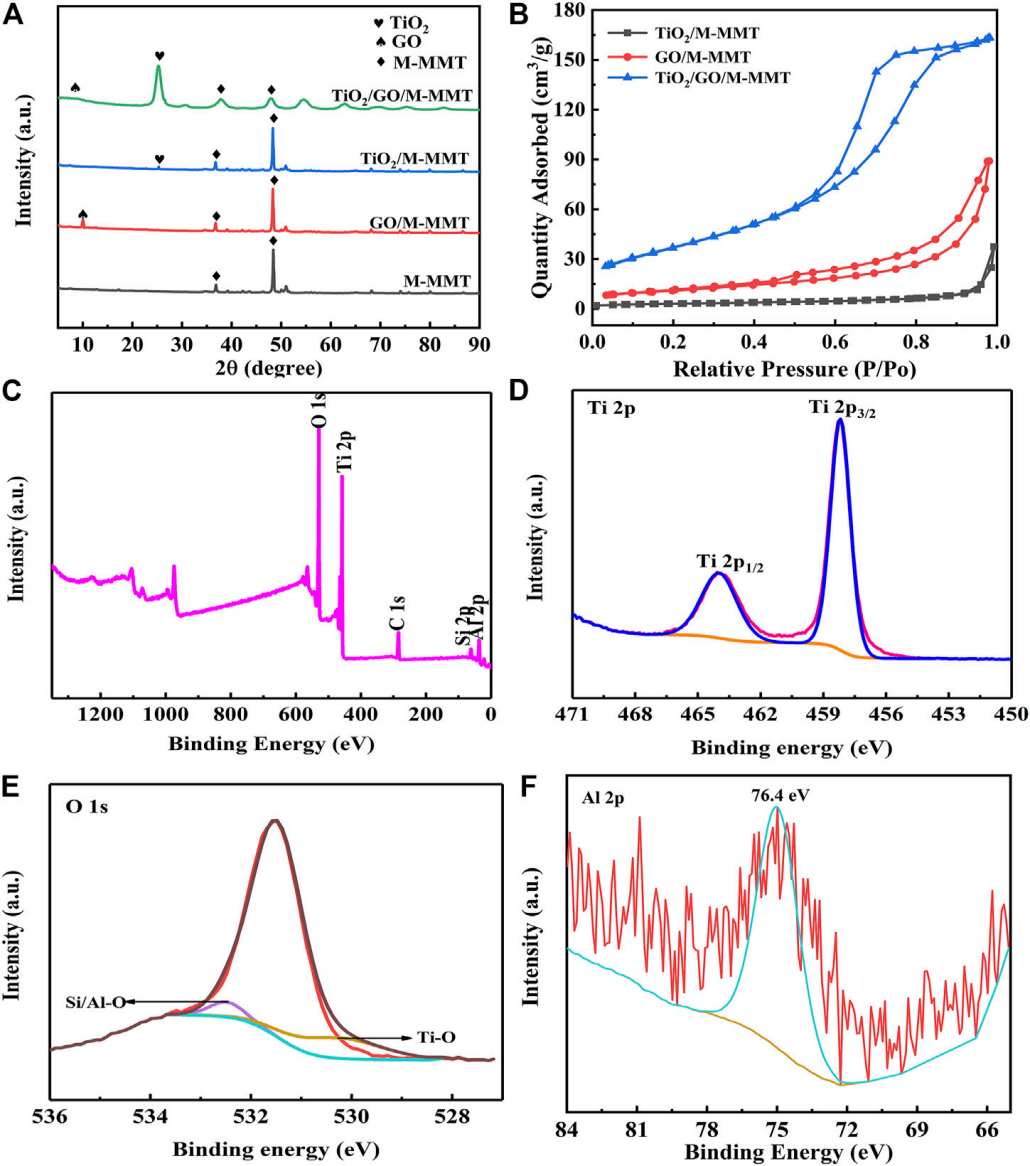
**(A)** X-ray diffraction patterns of different specimen with M-MMT, GO/M-MMT, TiO_2_/M-MMT and TiO_2_/GO/M-MMT; **(B)** Nitrogen adsorption and desorption curve of different nanocomposite; **(C)** XPS full spectrum of TiO_2_/GO/M-MMT and high resolution XPS spectrum of **(D)** Ti 2p; **(E)** O 1s; **(F)** Al 2p.

The XRD patterns obtained for M-MMT, GO/M-MMT, TiO_2_/M-MMT, and TiO_2_/GO/M-MMT are shown in Figure 3A. The XRD patterns obtained for M-MMT display a weak diffraction peak near 7°, which corresponds to the (100) characteristic diffraction peak of M-MMT. In addition to the characteristic M-MMT peaks, the GO/M-MMT spectrum displays an additional strong diffraction peak at 10°, which corresponds to the (002) characteristic peak of GO. A weak diffraction peak is observed in the TiO_2_/M-MMT spectrum at 25.5°, corresponding to the (101) peak of TiO_2_. Diffraction peaks corresponding to GO and TiO_2_ are observed in the spectrum of TiO_2_/GO/M-MMT. The diffraction-peak intensity corresponding to TiO_2_ is higher than that corresponding to TiO_2_/M-MMT, In TiO_2_/M-MMT, the ratio of M-MMT is much higher than that of TiO_2_, the grains are closely arranged in the same direction and the crystallinity is better in the diagram, which makes the intensity of the M-MMT diffraction peak is higher than that of others. The higher diffraction peak shows that the TiO_2_ diffraction peak is very small. In TiO_2_/GO/M-MMT composites, the addition of GO makes it enter into the layered M-MMT layers, which changes the distance between layers, so that the diffraction peak of M-MMT becomes shorter and wider, and the intensity of TiO_2_ diffraction peak increases after data normalization; however, the peak at 25.5° shifts to a lower angle. Because the M-MMT load is a lamellar structure, the layer spacing becomes wider because the addition of TiO_2_ and GO enter the interlayer. According to the Bragg equation 2dsinθ = nλ, the value of d increases and the value of *θ* decreases. The diffraction peak of M-MMT is relatively weak, indicating that TiO_2_ is attached to the surface of M-MMT. In addition, the diffraction peak of the composite is broadened. This could be because TiO_2_ and GO entered the M-MMT interlayer *via* intercalation.

Figure 3B shows the specific surface area of the GO/M-MMT, TiO_2_/M-MMT, and TiO_2_/GO/M-MMT nanocomposites. The specific surface area of TiO_2_/M-MMT, GO/M-MMT, and TiO_2_/GO/M-MMT nanocomposites were measured as 12 m^2^/g, 42 m^2^/g, and 117 m^2^/g, respectively. The specific surface area of the TiO_2_/GO/M-MMT composite is approximately ten and three times higher than that of TiO_2_/M-MMT and GO/M-MMT, respectively. The large specific area opened more number of ion-transportation channels, resulting in a faster electron transport rate and more number of adsorption active sites, thereby improving the photocatalytic performance. Supplementary Figure S2 shows the pore-size distributions of the GO/M-MMT, TiO_2_/M-MMT, and TiO_2_/GO/M-MMT nanocomposites, which have average pore sizes of 6.7, 9.2, and 13.4 nm, respectively. The average pore size of the TiO_2_/GO/M-MMT nanocomposite is the highest, indicating that the ion transport channels become wider, resulting in higher electron transport rates and photocatalytic degradation rates (Kočí et al., 2014; Mottola et al., 2022).

The characteristic peaks of Al, Si, C, Ti, and O are observed in the XPS full spectrum of the TiO_2_/GO/M-MMT nanocomposite (Figure 3C). The peaks of 458.6 and 464.4 eV observed in the Ti 2p high-resolution spectrum (Figure 3D) can be attributed to Ti 2p_3/2_ and Ti 2p_1/2_, respectively, indicating that Ti bonded to oxygen and exhibited a +4 valence in the composites. The O 1s peak is separated and fitted as shown in Figure 3E. The peaks at 531.5, 532.0, and 532.5 eV correspond to the O–O, Ti–O, and Si/Al–O bonds, respectively. The characteristic peak at 74.6 eV in the Al 2p XPS profile (Figure 3F) represents the Al–O bond in M-MMT.

**FIGURE 4:**
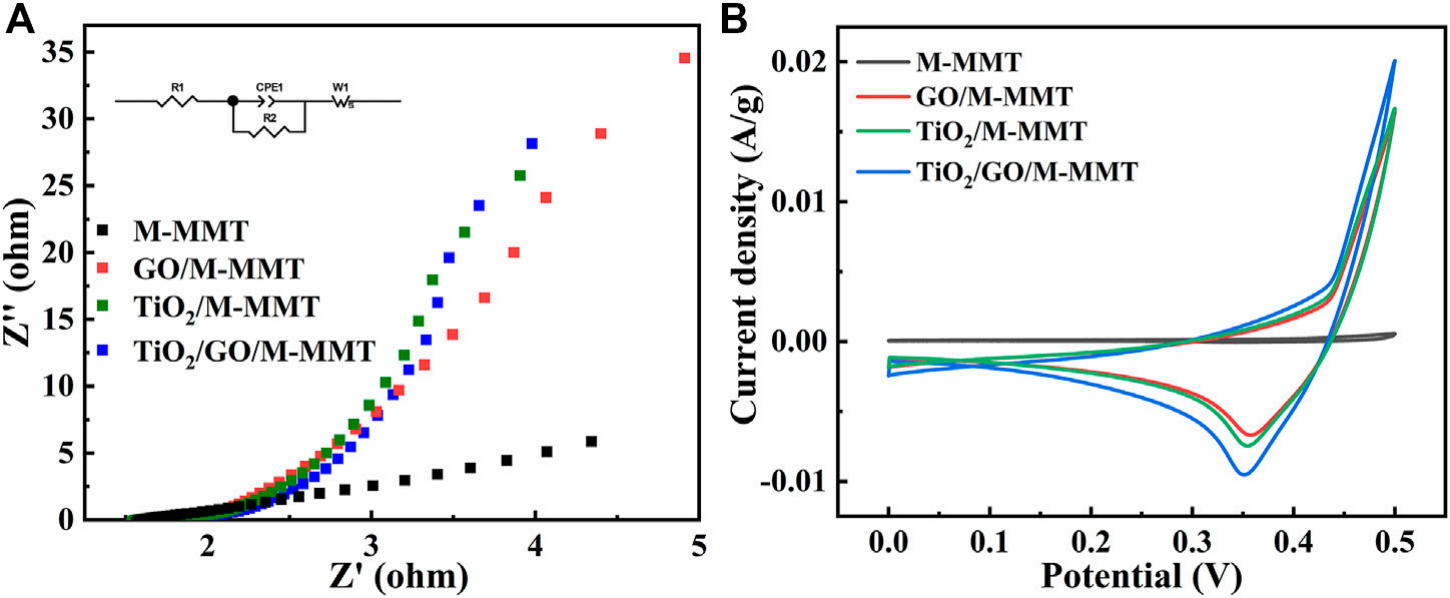
**(A)** EIS Nynquist plots of M-MMT, GO/M-MMT, TiO_2_/M-MMT and TiO_2_/GO/M-MMT nanocomposite; **(B)** Current−potential plots of different nanocomposite.

Figure 4A shows EIS Nynquist plots of different composites. Because M-MMT is a natural mineral material, the conductivity is weak and the slope is low. With the addition of GO and TiO_2_, the conductivity is improved, and the slope is increased. In TiO_2_/GO/M-MMT nanocomposites, due to the synergistic effect of GO and TiO_2_, the separation of electron-hole pairs is accelerated and the lifetime of photoinduced carriers is prolonged, thus enhancing the photocatalytic activity. The conductivity is enhanced, and the slope value is gradually close to 1, showing strong conductivity. Figure 4B shows the current-potential curves of different composites. The area enclosed by the CV curve is the activity of the material, and the more active the photocatalytic performance is. The CV curve of M-MMT in the diagram is almost a straight line, and the macroscopic performance is poor catalytic performance. In GO/M-MMT and TiO_2_/M-MMT, with the addition of GO and TiO_2_ to M-MMT, the activity gradually increased and the reduction peak appeared. In TiO_2_/GO/M-MMT, the area is the largest, the position of the reduction peak is the lowest, and the activity is the best, indicating that its photocatalytic performance is the best.

**FIGURE 5:**
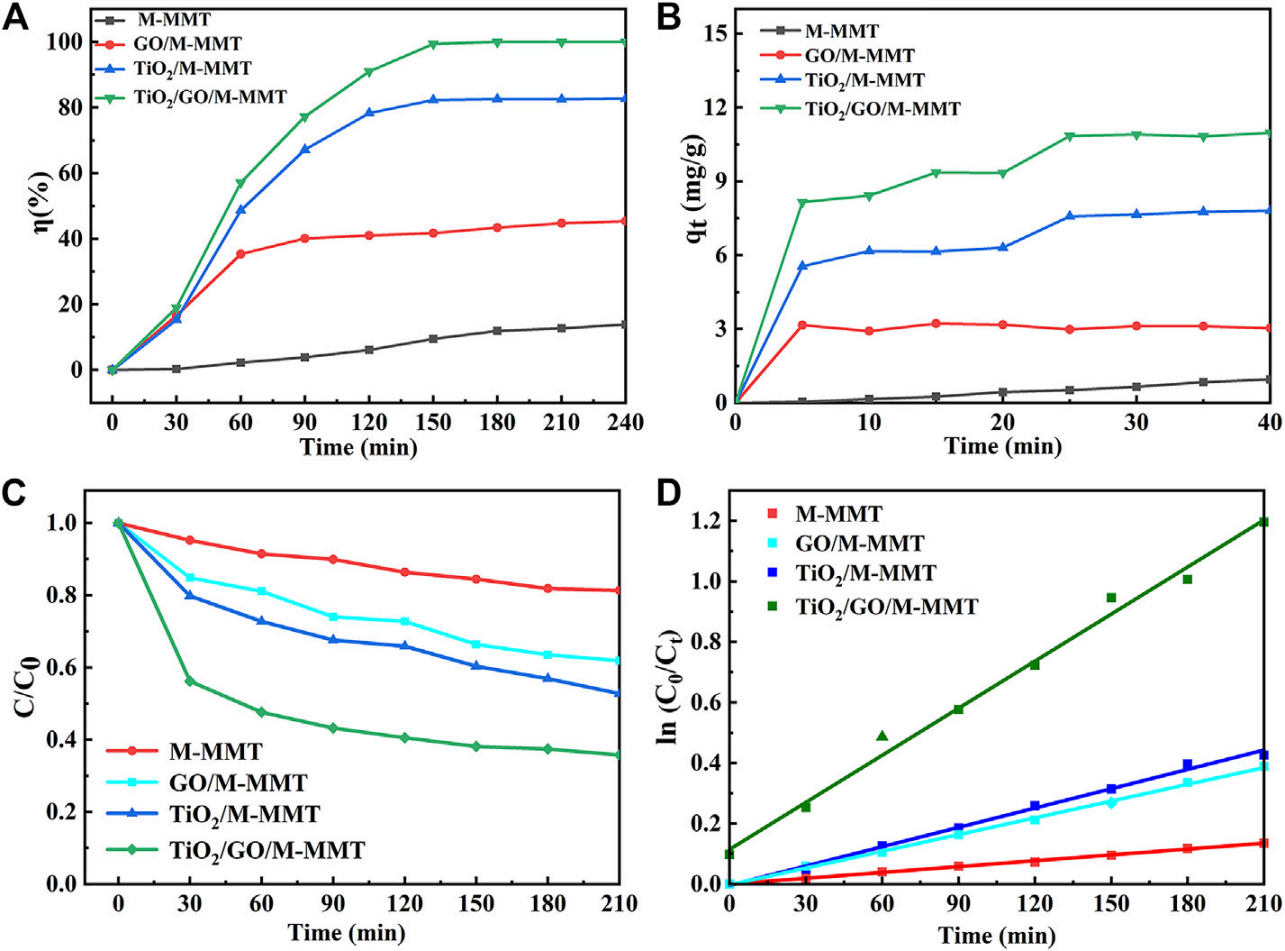
Photocatalytic performance curve of different photocatalyst. **(A)** The photocatalytic degradation rate curve; **(B)** The adsorption capacity curve; **(C)** Photodegradation concentration curve of MO; **(D)** Pseudo-first-order kinetic equation fitting diagram of different nanocomposite.

Figure 5A shows the rate of degradation of MO using the photocatalysts prepared in the study. The degradation test conducted for 240 min reveals that the rate of degradation of MO using M-MMT is only 15%. As the amounts of GO and TiO_2_ increase, the rate of degradation of MO increases to 41% and 79%, respectively. With the TiO_2_/GO/M-MMT nanocomposite, the rate of degradation of MO is 99%, which is twice as high as that achieved with GO/M-MMT. These results indicate that the TiO_2_ component plays an important role in the MO degradation process.

Figure 5B shows the adsorption performances of the M-MMT, GO/M-MMT, TiO_2_/M-MMT, and TiO_2_/GO/M-MMT nanocomposites for MO. After the visible-light illumination, the adsorption performance of the composites TiO_2_/M-MMT and GO/M-MMT are superior to that of M-MMT. The TiO_2_/GO/M-MMT nanocomposite exhibits the highest adsorption capacity. The higher photocatalytic activity of the TiO_2_/GO/M-MMT nanocomposite is attributed to the presence of the M-MMT component, which improves the dispersion ability of the photocatalyst and enhances the absorption capacity for photons and MO molecules in the composites. Further, the addition of TiO_2_ and GO extends the response range for visible light, thereby effectively reducing the recombination rate of electric carriers (Lin et al., 2020; Xiang et al., 2021).

The kinetics of the photocatalytic degradation was studied further to investigate the mechanism of the photocatalytic degradation. A pseudo-first-order kinetic equation is given in the following equation (Eq. 3) (Sharma et al., 2018).

$$\ln\left(C_{0}/C_{t}\right)=kt$$ (3)

where k is the quasi-first-order reaction rate constant (min-1) and t is the irradiation time (min).

Figure 5C shows the change in the concentration of MO after adsorption using the different composites. For all composites, the concentration of MO decreases after adsorption. The change in the concentration of MO observed after adsorption achieved with the M-MMT photocatalyst is small; however, the concentration considerably decreases after adsorption achieved with TiO_2_/GO/M-MMT. The TiO_2_/GO/M-MMT system can produce higher yield of •O^2-^, which generate more other active species and sites. Accordingly, the TiO_2_/GO/M-MMT composite had a high photocatalytic performance with excellent stability and can be recommended for removing the antibiotic compounds. The concentration of MO with different adsorption times is fitted for different photocatalysts, as shown in Figure 5D. The k value estimated for M-MMT is observed to range from minimum to zero, indicating a weak response to visible light. The k value for the TiO_2_/M-MMT nanocomposite is higher than that of the GO/M-MMT nanocomposite, indicating that the response time of TiO_2_/M-MMT to visible light is longer than that of GO/M-MMT. The degradation rate can be significantly increased using a combination of the three components (the k value increases). GO can reduce the space charge region of TiO_2_ and induce its electric field to separate photogenerated carriers effectively, which increases the photocatalytic activity. However, when the space charge region becomes too narrow, the dopant concentration increases and the recombination of photogenerated carriers becomes faster (Lincho et al., 2021; Olea et al., 2021; Li et al., 2022b).

**FIGURE 6:**
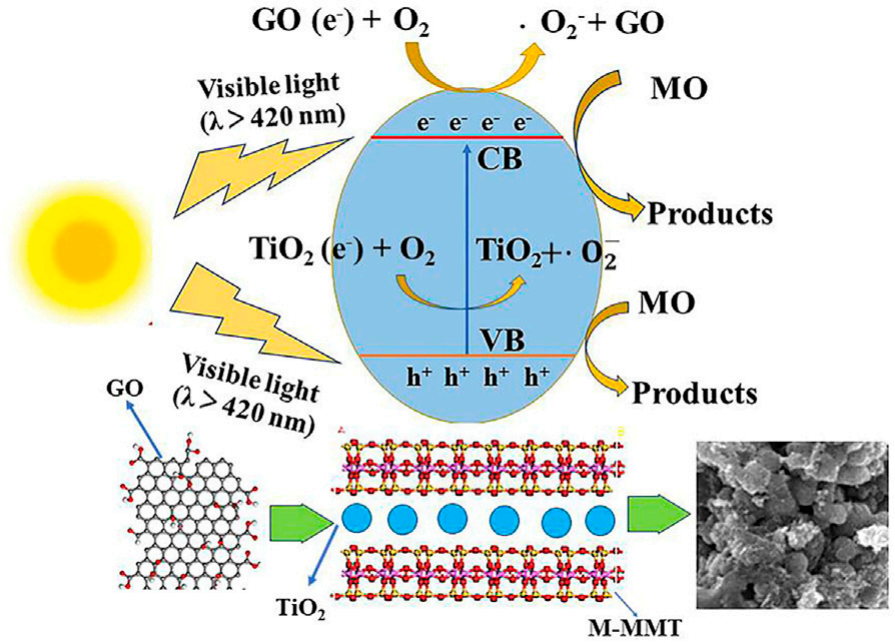
Photocatalytic degradation mechanism of MO by TiO_2_/GO/M-MMT.

According to experimental results explained above, the mechanism of the photocatalytic degradation of MO using TiO_2_/GO/M-MMT is proposed, as shown in Figure 6. When the MO dyes are irradiated with visible light, the MO dyes become photosensitized and initiate the photocatalytic process. Visible light is absorbed by the photosensitized MO dye molecules, which excites the MO-dye electrons to a higher energy level. Furthermore, the light-triggered electrons are transported from the high-energy state to the conduction band of TiO_2_, and MO is degraded by the active TiO_2_. Moreover, some electrons are transferred from TiO_2_ to GO. Because the two-dimensional π-conjugated structure in GO is the electron acceptor, this special structure can effectively suppress the recombination between charges and carriers. The recombination between the light-triggered electrons and oxygen produces superoxide radicals (•O^2-^) (Tahir, 2017). MO dye molecules are oxidized by these superoxide radicals to produce CO_2_, H_2_O, and intermediates. In addition, the layered structures of M-MMT enhance the adsorption capacity of the composites, which improve the photocatalytic performance (Chen et al., 2018; Yang et al., 2019; Foroutan et al., 2020; Mallik et al., 2021).

## 4 Conclusion

To promote the uniform dispersion of nano-TiO_2_ and improve the adsorption capacity of the photocatalyst, TiO_2_/GO/M-MMT nanocomposites were prepared. The main conclusions are as follows:

1) The interlayer space in the nanocomposites increased with the addition of GO, providing more space for TiO_2_ to enter the interlayer. The uniform nano-sized TiO_2_ particles were distributed in the interlayer and on the surface of the TiO_2_/GO/M-MMT nanocomposite, which formed ideal nanostructures.
2) The photocatalytic degradation rate of MO by the TiO_2_/GO/M-MMT nanocomposite was up to 99.3%. The nanocomposite exhibited a better adsorption performance, which conformed to the pseudo-first-order kinetic equations.

## Data Availability

The original contributions presented in the study are included in the article/Supplementary Material, further inquiries can be directed to the corresponding author.
