# Supplementary material for: Novel TiO2/GO/M-MMT nano-heterostructured composites exhibiting high photocatalytic activity
Source: Front Chem. 2023 Mar 9;11:1113186. doi: 10.3389/fchem.2023.1113186 (PMC10033540; doi:10.3389/fchem.2023.1113186)
Supplement: Supplementary file 1 [file DataSheet1.docx]

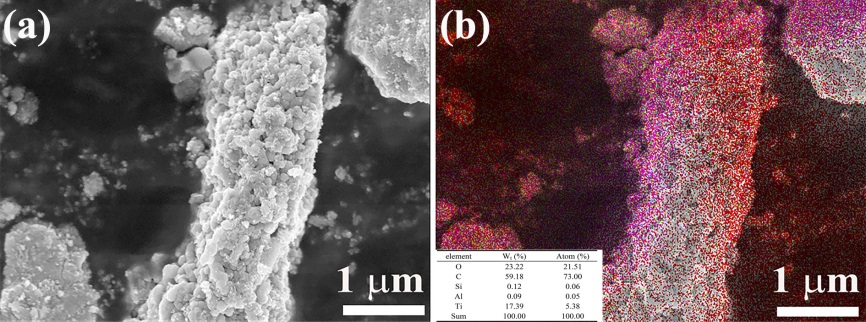


Supplement figure S1 EDS-Mapping of TiO_2_/GO/M-MMT nanocomposite; Inset table in Fig. S1(b) was the element content distribution of TiO_2_/GO/MMT composite.


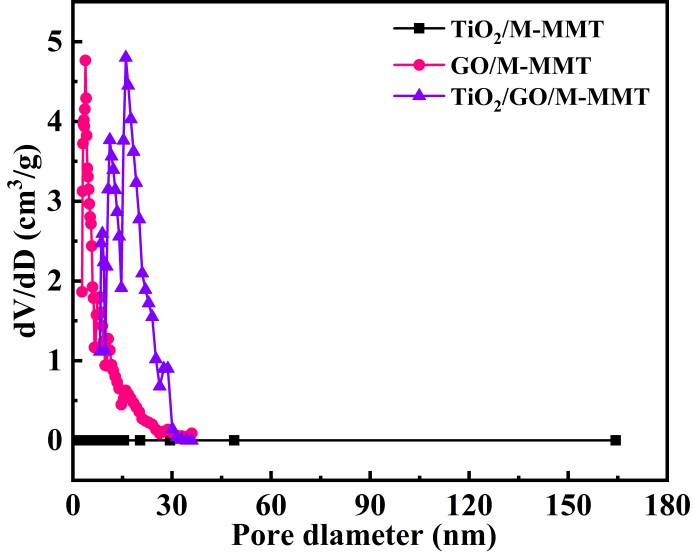


Supplement figure S2 Pore size distribution of GO/M-MMT, TiO_2_/M-MMT and TiO_2_/GO/M-MMT nanocomposite.
